# Validation of a murine proteome-wide phage display library for identification of autoantibody specificities

**DOI:** 10.1172/jci.insight.174976

**Published:** 2023-12-08

**Authors:** Elze Rackaityte, Irina Proekt, Haleigh S. Miller, Akshaya Ramesh, Jeremy F. Brooks, Andrew F. Kung, Caleigh Mandel-Brehm, David Yu, Colin R. Zamecnik, Rebecca Bair, Sara E. Vazquez, Sara Sunshine, Clare L. Abram, Clifford A. Lowell, Gabrielle Rizzuto, Michael R. Wilson, Julie Zikherman, Mark S. Anderson, Joseph L. DeRisi

**Affiliations:** 1 Department of Biochemistry and Biophysics,; 2 Diabetes Center, School of Medicine,; 3 Biological and Medical Informatics Program,; 4 Weill Institute for Neurosciences, Department of Neurology, School of Medicine,; 5 Division of Rheumatology, Rosalind Russell and Ephraim P. Engleman Rheumatology Research Center, Department of Medicine, and; 6 Department of Laboratory Medicine, UCSF, San Francisco, California, USA.; 7 Human Oncology & Pathogenesis Program and Department of Pathology & Laboratory Medicine, Memorial Sloan Kettering Cancer Center, New York, New York, USA.; 8 Chan Zuckerberg Biohub, San Francisco, California, USA.

**Keywords:** Autoimmunity, Immunology, Adaptive immunity, Antigen, Autoimmune diseases

## Abstract

Autoimmunity is characterized by loss of tolerance to tissue-specific as well as systemic antigens, resulting in complex autoantibody landscapes. Here, we introduce and extensively validate the performance characteristics of a murine proteome-wide library for phage display immunoprecipitation and sequencing (PhIP-seq) in profiling mouse autoantibodies. This library was validated using 7 genetically distinct mouse lines across a spectrum of autoreactivity. Mice deficient in antibody production (*Rag2^–/–^* and μMT) were used to model nonspecific peptide enrichments, while cross-reactivity was evaluated using anti-ovalbumin B cell receptor–restricted OB1 mice as a proof of principle. The PhIP-seq approach was then utilized to interrogate 3 distinct autoimmune disease models. First, serum from *Lyn^–/–^* *IgD*^+/–^ mice with lupus-like disease was used to identify nuclear and apoptotic bleb reactivities. Second, serum from nonobese diabetic (NOD) mice, a polygenic model of pancreas-specific autoimmunity, was enriched in peptides derived from both insulin and predicted pancreatic proteins. Lastly, *Aire^–/–^* mouse sera were used to identify numerous autoantigens, many of which were also observed in previous studies of humans with autoimmune polyendocrinopathy syndrome type 1 carrying recessive mutations in AIRE. These experiments support the use of murine proteome-wide PhIP-seq for antigenic profiling and autoantibody discovery, which may be employed to study a range of immune perturbations in mouse models of autoimmunity profiling.

## Introduction

Autoimmune diseases arise from a complex breakdown in immune tolerance and are frequently characterized by the presence of autoantibodies and autoreactive T cells. Autoimmunity spans a breadth of different clinical subtypes and patterns, with almost any organ system or tissue being susceptible. Defining the specificity and origins of the autoimmune response is key for developing methods to diagnose, prevent, and treat this family of diseases. On a mechanistic level, a key tool for unraveling autoimmunity has been the use of mouse models of human autoimmune diseases. A combination of autoimmune-susceptible mouse strains or mouse lines with models of human genetic defects has played a key role in our understanding in the pathogenesis of an array of autoimmune diseases. For example, use of genetically altered mice has allowed for our understanding of how the monogenic autoimmune diseases autoimmune polyendocrine syndrome type 1 (APS1) (1) and immune dysregulation, polyendocrinopathy, enteropathy, X-linked syndrome (IPEX) (2, 3) are linked to defects in thymic central tolerance or the function of T regulatory cells, respectively. Given the highly controlled nature of mouse modeling for both environmental and genetic influences, it remains an essential tool for dissecting autoimmunity. An important aspect to this work is defining the autoimmune response in these mouse models. In this regard, a typical approach has been to search for autoantibodies from affected mice in targeted assays such as Western blotting and indirect immunofluorescence. Recently, there has been rapid development of new approaches to identifying autoantibody specificities that broadly cover the entire proteome in the human setting (4–9). Thus, a similar approach in a mouse model could serve as an important method to further define and unravel autoimmunity.

Phage display immunoprecipitation and sequencing (PhIP-seq) is a powerful tool to identify antibody targets, originally described by Larman, Elledge, and colleagues (4, 10). Since 2011, it has been used to discover novel antibody autoreactivities in a wide range of human syndromes, including paraneoplastic diseases (6, 7) and inborn autoimmune syndromes (8, 9, 11). In PhIP-seq, libraries of long oligonucleotides encoding overlapping peptides are synthesized as DNA oligomers and cloned into the T7 phage genome. These libraries are expressed fused to gene 10 of the surface-exposed capsid protein on lytic T7 phage and used as bait for antibodies in patient sera. Complex, multiantigen immunoprecipitants are deconvoluted by sequencing the enriched phage-encoded peptides to identify multiple antibody targets in a single reaction.

Due to the programmable nature of PhIP-seq, any proteome may be comprehensively encoded in a phage library in principle. Library designs may also be highly customized, including coverage of specific protein isoforms, putative coding regions, and other features. Here, we present the construction and validation of a murine proteome-wide PhIP-seq library, based on the GRCm38.p5 *Mus musculus* genome, composed of over 480,000 peptides, representing over 76,000 protein sequences. Taking advantage of genetic manipulations available in the mouse model, library performance was evaluated across 7 mouse strains, including *Rag2^–/–^*, those expressing genetically modified immunoglobulin M transmembrane domains (μMT mice), C57BL/6J (wild-type strain, B6), OB1, *Lyn^–/–^*, polygenic nonobese diabetic (NOD), and *Aire^–/–^*. Mice lacking mature B cells (*Rag2^–/–^* and μMT) were used to determine proteome-wide background binding in immunoprecipitations (IPs), while serum from B6, OB1, *Lyn^–/–^*, NOD, and *Aire^–/–^* mice was used to identify strain-specific autoreactivities. Building on our previous identification of autoantibodies against perilipin-1 (Plin1) in *Aire^–/–^* mice (9), we identified the binding epitope of these antibodies and demonstrate their relationship to immune cell infiltrates in adipose tissue in affected mice. Taken together, these results demonstrate the utility of the approach for a broad assessment of the array of autoimmune specificities in various mouse models.

## Results

### Design and construction of murine proteome-wide library.

**Figure 1:**
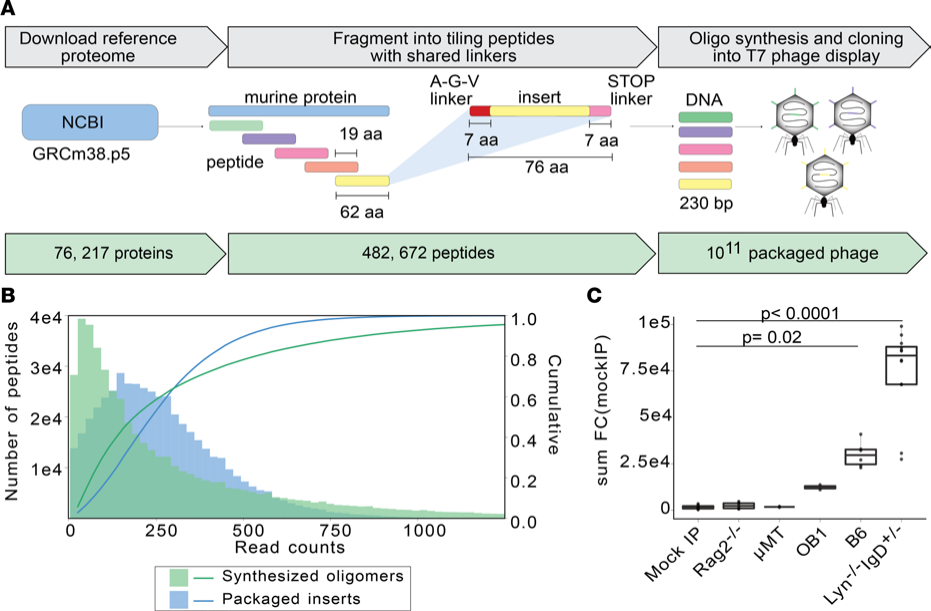
Design and validation of murine PhIP-seq library. (**A**) GRCm38.p5 annotated proteins were downloaded from Refseq and 62–amino acid (62-aa) tiles were chosen to cover the 76,217 proteins with 482,672 peptides with a 19-aa overlap. The tiles contained necessary cloning sites for expression in a T7 phage display system. (**B**) Representation of designed oligonucleotides after oligonucleotide synthesis and cloning. (**C**) Sum of all fold changes (FCs) above the mean read counts in mock IP in each experimental sample (mock IP) or mouse strain (*Rag2^–/–^*, μMT, OB1, B6, and *Lyn^–/–^* *IgD*^+/–^) by PhIP-seq. Exact *P* value is reported, and each dot corresponds to a mouse or mock-IP replicate. Kruskal-Wallis test with Tukey’s HSD post hoc test.

To construct a *Mus musculus* proteome-wide library, the GRCm38.p5 reference proteome sequences, including all isoforms, were downloaded from the NCBI and divided into 62–amino acid peptide tiles with 19–amino acid overlaps (Figure 1A). The library was supplemented with several positive and negative control peptides, including those derived from human glial fibrillary acid protein (GFAP), human tubulin, GFP, and others. The resulting library of 482,672 peptides was synthesized (Agilent) as a DNA oligomer pool (Figure 1A) and cloned into T7 phage fused in frame with gene 10, which encodes the capsid protein of T7 phage. The complete peptide design file and further details are freely available as a companion to this manuscript on protocols.io (see Methods). The synthesized oligomer library and the packaged library were sequenced by next-generation sequencing with 173 and 79 million paired-end 147–base pair reads on an Illumina NovaSeq 6000, respectively, which resulted in an approximate 360× and 163× coverage of the library, respectively. Alignment of the reads from sequencing of the pooled DNA oligomer library yielded 89.2% identical matches, with greater than 99.9% of all the expected peptides represented. Sequencing of the packaged T7 phage library yielded an alignment rate of 78.9%, yet representation remained high at greater than 99% of the expected peptides (Figure 1B).

### Evaluation of library performance and background binding in Rag2^–/–^ and μMT mice.

The performance of the packaged library was first benchmarked utilizing a commercial antibody with a known specificity. Previously, we have utilized a commercial anti-GFAP polyclonal antibody as a positive control for human PhIP-seq libraries, due to its consistent IP performance (8, 11–13). The murine PhIP-seq library contains both human and mouse GFAP sequences and binding to sequences of both species was expected with the commercial antibody. Antibody bound to a mix of protein A and protein G magnetic beads was used to IP phage from the murine library, followed by either sequencing or further amplification in *Escherichia coli*. As observed previously with the human PhIP-seq library (8, 13), additional rounds of phage IP followed by amplification in *E*. *coli* resulted in increased enrichment of the target sequences. Using 2 rounds, approximately 20% of resulting phage encoded peptides derived from either human *GFAP* or mouse *Gfap* (Supplemental Figure 1A; supplemental material available online with this article; https://doi.org/10.1172/jci.insight.174976DS1). Using 3 rounds, the enrichment approached 50%, or 1 × 10^8^–fold greater than the amount of the same phage in the starting library (Supplemental Figure 1A). Given the significant enrichment of Gfap peptides relative to nonspecific peptides, all subsequent experiments utilized 3 rounds of IP and amplification.

The library was next evaluated across 5 mouse strains on the B6 background. Two strains (*Rag2^–/–^* and μMT) lacking IgG (14, 15) were utilized to evaluate background binding. When compared with a mock IP control lacking serum, sera from both *Rag2^–/–^* and μMT mice failed to significantly enrich phage from the murine library, as expected (Figure 1C and Supplemental Figure 1B). At the individual peptide level, fewer than 10 peptides were consistently enriched (at least 2 of 3 mice) by sera from either mouse strain, presumably through nonspecific interactions (Supplemental Figure 1C). For subsequent experiments, the mean frequency of each phage across mock IP, *Rag2^–/–^*, and μMT was determined and used to calculate fold-change and *z* scores for experimental samples (murine background model, MBM).

B cells in OB1 mice harbor physiological B cell receptor (BCR) rearrangements in the IgH and Igκ loci that encode for a clonal IgG1 receptor with specificity for the chicken ovalbumin (OVA) protein; thus, their sera contains predominantly high-affinity anti-OVA IgG1 antibodies (16). Unlike *Rag2^–/–^* and μMT mice, sera from OB1 mice yielded a moderate amount of enrichment consistent with the restricted B cell repertoire of this genetic strain (Figure 1C and Supplemental Figure 1B). In contrast, sera from wild-type B6 mice yielded significant enrichment, with 3.2-fold more enrichment than OB1 sera (Figure 1C and Supplemental Figure 1B). Finally, *Lyn^–/–^* *IgD*^+/–^ mice were also examined, which exhibit polyclonal B cell activation and develop lupus-like disease (17, 18). Consistent with a greater degree of autoreactivity in these mice, *Lyn^–/–^* *IgD*^+/–^ sera yielded the largest number of significantly enriched peptides, with higher fold-change than sera from wild-type B6 mice (Figure 1C and Supplemental Figure 1B).

### Proteome-wide PhIP-seq identifies OB1 reactivity to known epitopes in mouse proteome.

**Figure 2:**
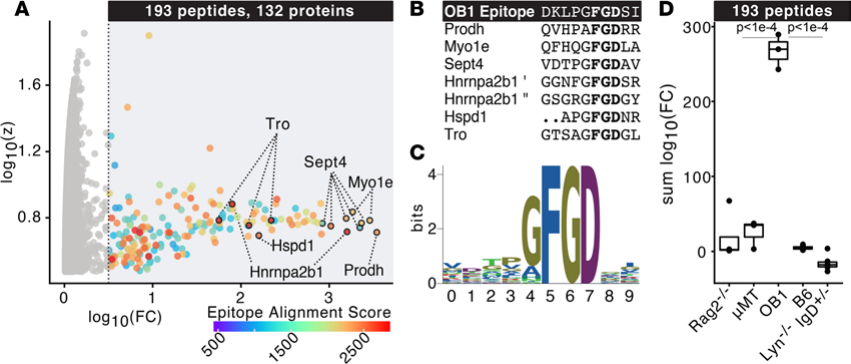
Identification of autoreactive epitopes recognized by ovalbumin-specific BCR-transgenic (OB1) mice. (**A**) Log_10_(fold change) and *z* score over murine background model (mean of *Rag2^–/–^*, μMT, and mock IP) of peptides enriched by PhIP-seq in OB1 mice colored by alignment score to known epitope and essential FGD motif. (**B**) Multiple sequence alignment of top OB1-enriched peptides. (**C**) Logo plot of multiple sequence alignment of 193 peptides enriched by OB1 sera. (**D**) Sum log_10_(fold change) over MBM of OB1 peptides enriched by sera from *Rag2^–/–^*, μMT, OB1, B6, or *Lyn^–/–^* *IgD*^+/–^ mice. Exact adjusted *P* value is reported, and each dot corresponds to 1 mouse. Kruskal-Wallis test with Tukey’s HSD post hoc test.

Previously, the recognition site for immunoglobulin binding to the OVA protein in the OB1 strain was determined as DKLPGFGDSI by alanine mutagenesis, where the Phe-Gly-Asp (FGD) sequence was essential for BCR binding (16). Although chicken OVA sequences were not included in this murine PhIP-seq library, over 2,000 peptides in the library contain sequence similarity (*P* < 0.0001; see Methods) to the OVA target sequence, and of those, 241 contain the critical FGD core sequence. Furthermore, an additional 2,028 peptides contain the core FGD, but lack similarity in the regions flanking this motif (see Methods and Supplemental Table 1). Using sera from OB1 mice, 193 peptides were enriched greater than 4-fold above the average of MBM, with a *z* score of greater than 3 (Figure 2A). To investigate similarity of enriched peptides to the known OB1 BCR recognition epitope, a scoring system for each peptide was developed using the sum of Smith-Waterman alignment scores to FGD across 4–amino acid sliding windows and weighted if an exact FGD match was present (see Methods). Most peptides enriched by OB1 sera exhibited high epitope similarity scores, indicating specific enrichment of peptides containing the known OB1 BCR binding site (Figure 2A). Visual inspection of the most enriched peptides revealed significant sequence similarity to the known OVA target sequence, including the core FGD (Figure 2B). We noted a Hnrnpa2b1 peptide containing 2 FGD repeats among the most highly enriched, indicating that valency within the peptide may contribute to greater enrichment.

Enriched peptides containing the core sequence were aligned, revealing over representation of N-terminal Gly (Figure 2C), suggesting that this residue may contribute to recognition. Overall, 80% of the significantly enriched peptides contain the FGD core recognition sequences. Compared with *Rag2^–/–^*, μMT, wild-type, and *Lyn^–/–^* *IgD*^+/–^ mice, these 193 significantly enriched peptides from the OB1 mice were highly specific to this strain (Figure 2D).

### Lyn^–/–^ IgD^+/–^ mice exhibit lupus-like autoantibody reactivity to nuclear and apoptotic antigens.

**Figure 3:**
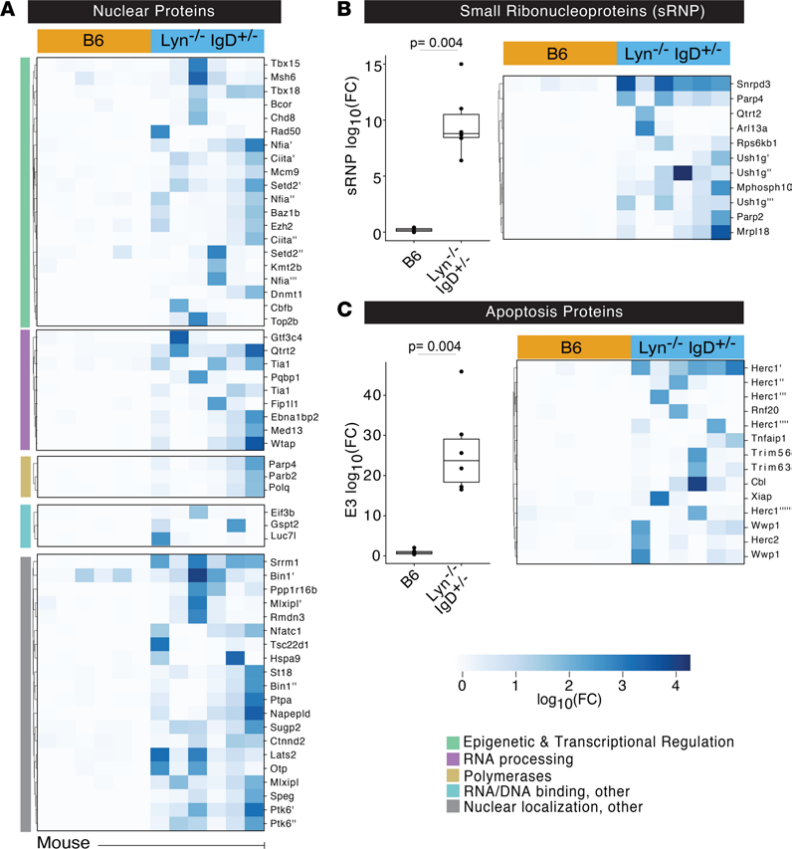
Autoreactivity to nuclear and apoptotic antigens in *Lyn^–/–^* *IgD*^+/–^ mice. (**A**) Heatmap of nuclear proteins enriched in *Lyn^–/–^* *IgD*^+/–^ versus wild-type B6 mice. (**B**) Sum log_10_(fold change) over mean background (left) and heatmap of log_10_(fold change) (right) of small ribonucleoproteins in B6 or *Lyn^–/–^* *IgD*^+/–^ mice. (**C**) Sum log_10_(fold change) over mean background (left) and heatmap of log_10_(fold change) (right) of E3 ubiquitin ligases in B6 or *Lyn^–/–^* *IgD*^+/–^ mice. Peptide enrichments were identified by PhIP-seq in **A**–**C**. Exact adjusted *P* value is reported, and each dot corresponds to 1 mouse. Kruskal-Wallis test with Dunn’s post hoc test.

Deficiency of the Src family kinase Lyn results in widespread autoantibody production and lupus-like disease because Lyn plays a nonredundant negative regulatory role downstream of the BCR by mediating immunoreceptor tyrosine-based inhibitory motif–dependent inhibitory signaling; such autoimmunity is accelerated in *Lyn^–/–^* *IgD^+/–^* mice due to impaired quiescence of autoreactive B cells regulated by *IgD* misexpression (17, 19). *Lyn^–/–^* *IgD*^+/–^ mice were selected for interrogation with the murine PhIP-seq library due to the profound defect in self-tolerance in this established model of lupus. Sera from *Lyn^–/–^* *IgD*^+/–^ mice (*n* = 6) and wild-type B6 mice (*n* = 6) were used to IP phage from the mouse PhIP-seq library. Sequencing libraries were prepared after 3 rounds of IP and amplification. The resulting read counts for each peptide were normalized to the total number of reads in each library and then averaged across all technical replicates for each mouse. High correlations (Pearson’s *r* > 0.75) were observed between technically replicated samples (Supplemental Figure 1D). A stringent set of requirements was then used to identify peptide enrichments that were specific to the mutant mice relative to controls. A total of 508 peptides, derived from 425 proteins (Supplemental Table 2 and Supplemental Figure 2A), had a fold-change of at least 2-fold, a *z* score of least 3, and were not enriched in any of the control B6 mice IPs. Approximately 37% of mutant-specific peptide enrichments corresponded to proteins known to be components of or related to nuclear proteins (Figure 3A), consistent with the high degree of anti-nuclear staining observed in these mice (Supplemental Figure 3) and in patients with lupus (20). To validate these findings, *Lyn*-specific PhIP-seq reactivities were compared to results from a 96-autoantigen array probed with sera from *Lyn^–/–^* mice (Supplemental Table 3). Among protein autoantigens represented in the array (*n* = 63), 32 proteins were significantly enriched in *Lyn^–/–^* IgG above wild-type controls (see Methods), 20 of which were also identified by PhIP-seq in *Lyn^–/–^* *IgD*^+/–^ mice (Supplemental Table 3). Orthogonally validated *Lyn*-specific reactivities were those to nuclear antigens such as nuclear proteins (SP100), complement (C1q), collagen (collagen VI) and laminin (Lama1), as well as additional nuclear proteins (Supplemental Figure 2, B–D, and Supplemental Table 3). In addition to small ribonucleoproteins (sRNPs), PhIP-seq identified proteins related to their generation that were not included in the targeted autoantigen array (Figure 3B). This overrepresentation of sRNP autoimmune targets in *Lyn^–/–^* *IgD*^+/–^ mice is consistent with prior reports that CD72-sRNP–negative signaling in B cells is *Lyn* dependent (21, 22). Identification of nucleic acid–associated autoantigens is also consistent with an obligate role for nucleic acid–sensing machinery in B cells in this mouse model (23) and in patients (24–26). PhIP-seq also identified 14 E3 ubiquitin ligases or E3 ubiquitin complex proteins and 50 additional proteins related to apoptosis (Figure 3C), which is notable given evidence of impaired clearance of apoptotic debris in lupus patients (27, 28). For example, reactivity to 5 peptides within *Herc1* and 1 peptide in *Herc2* were specifically enriched in *Lyn^–/–^* *IgD^+/–^* mice. *Herc1* and *Herc2* encode large ubiquitin ligases that regulate apoptosis via interaction with tumor suppressor proteins such as c-Raf (29) and p53 (30), respectively.

### Autoreactivities in NOD versus B6 mouse strains.

The murine PhIP-seq library was next used to interrogate autoantibody profiles in polygenic NOD mice (*n* = 9) and B6 mice (*n* = 11). NOD mice were chosen due to their predisposition to spontaneous autoimmunity that includes autoimmune diabetes. These mice carry an I-Ag7 MHCII haplotype that contributes to the generation of autoreactive T cells (31–34) and the development of autoantibodies with age, including those targeting insulin in older mice (35, 36). Using a *z*-score threshold of 3 relative to the MBM, modest but significant enrichment of peptides derived from mouse insulin (Ins1) and insulin-related proteins (Irs4, Insrr, Ide, and Insm1) was observed (Supplemental Figure 4, A and B) in NOD mice, but not in B6 mice.

Using the stringent enrichment criteria established for the *Lyn^–/–^* *IgD*^+/–^ experiment, a total of 314 putative autoantigens were identified (Supplemental Table 4). These included proteins expressed by pancreatic islet cells (Ptch1, Disp1, Pck1, and Cacna1e) as well as proteins known to be associated with diabetes mellitus susceptibility or glucose metabolism (Adcy8, Perm1, Sorbs1, and Pla2g6; Supplemental Figure 4, B and C). NOD mice develop additional autoimmune phenotypes such as Sjorgen’s (affecting salivary and lacrimal glands) (37), prostatitis (affecting seminal vesicle and prostate) (38), and respiratory infiltrates in older mice (39). Autoantigens affecting these tissues were also observed in NOD mice (Supplemental Figure 4C). For example, seminal vesicle proteins Svs3 and Svs4 were enriched in male NOD mice (Supplemental Figure 4C), consistent with reports that antibodies against seminal vesicle proteins are associated with lymphocytic infiltration of the prostate (40).

### Identification of autoreactivity and epitope mapping of anti-Plin1 antibodies in Aire^–/–^ mice.

We next investigated mice lacking the transcriptional regulator Aire, which drives expression of tissue-specific antigens in the thymus and is required for selection of the self-tolerant T cell repertoire (1). *Aire^–/–^* mice represent a severe form of autoimmunity due to a lack of central tolerance and this is accelerated on the NOD background. In humans, mutations in the AIRE transcription factor result in autoimmune polyendocrine syndrome type 1 (APS1), a rare monogenic disease in which patients develop multiorgan autoimmune pathologies due to autoreactive T cells and high-affinity tissue-specific B cells (41). Using a human proteome-wide PhIP-seq library, our lab has previously characterized APS1 patient sera and identified a wide array of novel autoreactive antigens (8, 9, 11).

**Figure 4:**
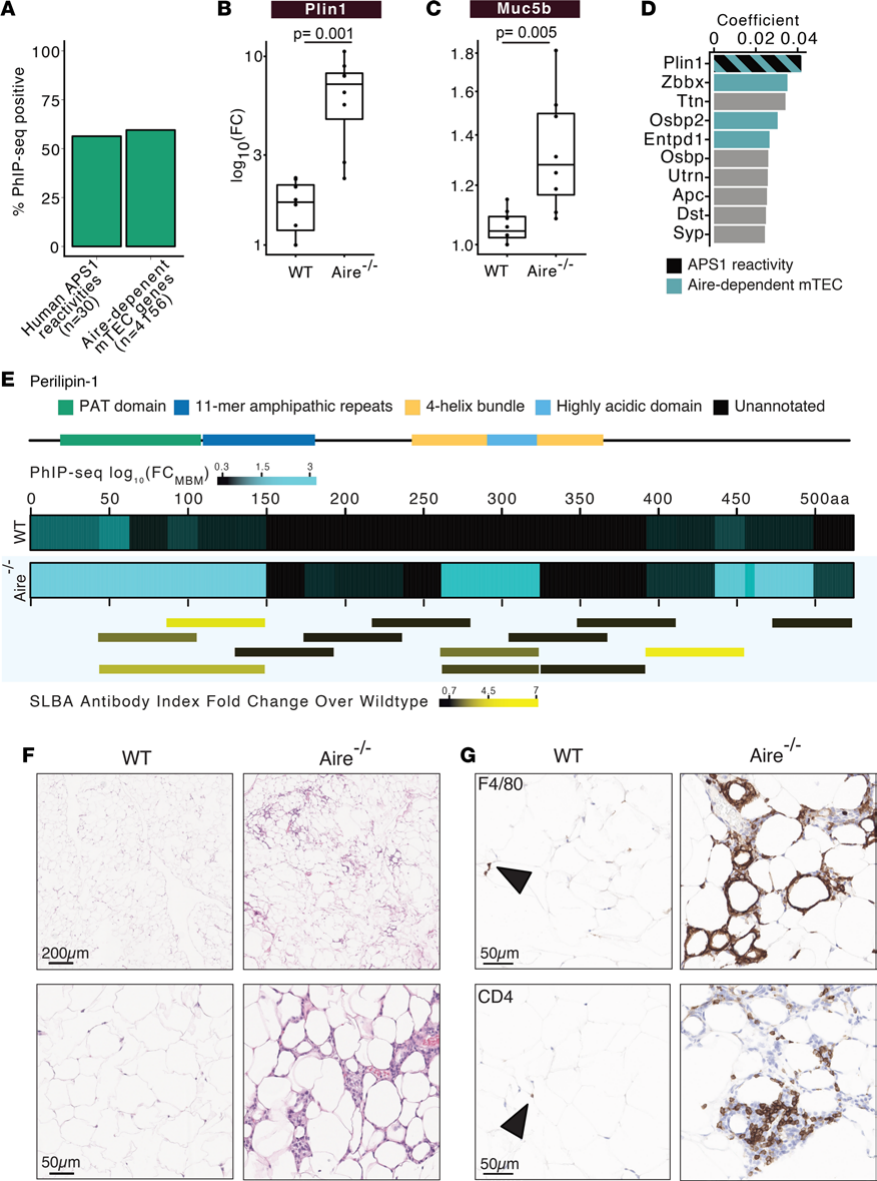
Autoreactivity in *Aire^–/–^* mice. (**A**) Percentage of peptides enriched in NOD.*Aire^–/–^* mice by PhIP-seq compared to orthologs of previously reported APS1 reactivities in humans (11) and genes under the control of Aire in mTEC (40). Sum log_10_(fold change) over murine background model of **B**. (**C**) Muc5b in *Aire^–/–^* versus control mice. (**D**) Logistical regression coefficients of top 10 proteins for classifying *Aire^–/–^* versus control mice colored by orthologous APS1 reactivity and/or Aire-dependent mTEC expression. (**E**) Heatmap of Plin1 positional sum log_10_(fold change) over background in *Aire^–/–^* or wild-type mice by PhIP-seq annotated with domain positions (top). Fold change of antibody index in *Aire^–/–^* over wild-type mice by SLBA (bottom). (**F**) Inguinal fat pads stained with H&E. Higher magnification is shown below. Scale bars: 200 μm (top) and 50 μm (bottom) (**G**) Immunohistochemistry of F4/80 (top) or CD4 (bottom) in inguinal fat pads in *Aire^–/–^* versus NOD mice. Arrowhead indicates positive cells. Scale bars: 50 μm. Peptide enrichments were identified by PhIP-seq in **A**–**E** and SLBA in **E** (bottom). Exact adjusted *P* value is reported, and each dot corresponds to 1 mouse. Kruskal-Wallis test with Dunn’s post hoc test.

To assess proteome-wide autoreactivity in *Aire^–/–^* mice, we performed PhIP-seq with sera of these mice on the NOD background and identified enriched peptides with a *z* score greater than 3 above MBM. Approximately 57% of the known autoimmune targets in human APS1 (*n* = 30 proteins) (11) were orthologous to putative autoreactive targets in *Aire^–/–^* mice and approximately 43% of genes with known *Aire*-dependent thymic expression (42) were among *Aire^–/–^*-specific reactivities by PhIP-seq (Figure 4A). Using the same stringent criteria as the *Lyn^–/–^* *IgD*^+/–^ experiment, serum from *Aire^–/–^* but not control mice enriched 927 peptides, representing 747 proteins, which was significantly more than *Lyn^–/–^* *IgD*^+/–^ mice (927 versus 494; Supplemental Table 5 and Supplemental Figure 5A). These included Plin1 and Muc5b (Figure 4, B and C), which were both detected in APS1 patients and their expression is dependent on *Aire* in the thymus. Using a 3-fold cross-validated logistical regression model trained on control and *Aire^–/–^* PhIP-seq data, *Plin1* was the top-ranked feature by the logistic regression coefficient (Figure 4D). Plin1 was previously identified as an autoimmune marker of generalized lipodystrophy in both mice and humans, including at least one patient with APS1 (9, 43).

Examination of the peptide-level enrichment data for Plin1 across all 8 *Aire^–/–^* mice revealed differential enrichment in the PAT domain, and to a lesser extent, the 11-mer amphipathic repeat region, and the highly acidic domain (Figure 4E). The PAT domain is highly conserved in the Plin family of proteins, yet other Plins (*Plin2*, *Plin3*, *Plin4*, and *Plin5*) did not exhibit enrichment in *Aire^–/–^* mice (Supplemental Figure 5B), suggesting that the epitope is specific to *Plin1*. Positional conservation analysis of the PAT domain across Plins showed greatest divergence in 2 regions corresponding to highest *Aire^–/–^* reactivity within Plin1 (Supplemental Figure 5C), plausibly explaining antibody specificity for Plin1 but not other members of the family.

To validate these findings, a split luciferase binding assay (SLBA) was developed by generating peptides (*n* = 13) tiling across Plin1 with a HiBiT tag that complexes with LgBiT to produce luminescence (Figure 4E and Supplemental Figure 6A). SLBA possess several advantages for rapid orthogonal validation of PhIP-seq results, including features of the luciferase immunoprecipitation system (LIPS) (44) and the radioligand binding assay (RLBA) (45). All Plin1 fragments generated at least 5-log greater luciferase signal than an in-frame stop codon construct when LgBiT and luciferase substrate was added (Supplemental Figure 6B). IPs were performed with tagged peptides and *Aire^–/–^* or control sera. Luciferase activity was measured after IP and fold-change over wild-type control mice was calculated for antibody indices using an anti-HiBiT antibody (see Methods). The most highly enriched SLBA fragments corresponded to the most enriched PhIP-seq peptides in the PAT domain, with the exception of 1 enriched fragment in a C-terminal unannotated region (Figure 4E).

As noted, anti-PLIN1 is a known a marker for acquired generalized lipodystrophy in humans (9). Generalized lipodystrophy is characterized by progressive loss of adipose tissue, panniculitis, and metabolic abnormalities (46). To investigate whether *Aire^–/–^* mice also exhibit similar features, gonadal fat pads were analyzed by H&E staining (Figure 4F). *Aire^–/–^* mice exhibited elevated cellular infiltration as compared with wild-type controls (Figure 4F and Supplemental Figure 7) and immunohistochemical analysis indicated the presence of macrophages (F4/80^+^ cells) and CD4^+^ T cells (Figure 4G). These data suggest that *Aire^–/–^*-driven autoimmune lipodystrophy in mice possesses similar features to its human disease counterpart, with Plin1 autoreactivity being the hallmark in both.

## Discussion

The PhIP-seq technique has been used extensively for the identification of autoreactivies in human sera and cerebrospinal fluid, including for the identification of novel autoimmune diseases (4–13). While the technique has proven useful in human clinical investigations, a parallel murine PhIP-seq library provides new avenues to investigate immune dysregulation and disease in a tractable model organism. Here, we describe the construction and validation of a proteome wide murine PhIP-seq library, composed of greater than 480,000 peptides, each 62 amino acids in length, spanning over 76,000 protein sequences. The design of short peptides of equal length across the proteome was chosen to capture antibody-epitope interactions and our bioinformatics analyses, performed on the peptide level, considers each peptide independently. While similar in design, this library includes approximately 27,000 additional protein sequences (including variants) compared with a previously published library (47). For any newly designed PhIP-seq library, it is essential to quantify the performance characteristics. Here, this murine PhIP-seq library was extensively validated using 7 different mouse strains, including 3 well-characterized autoimmune models, as well as wild-type, OB1, and mature B cell–deficient mice (*Rag2^–/–^* and μMT). The murine PhIP-seq library exhibited robust performance with control antibody and murine sera, lending further support for PhIP-seq as a reliable and sensitive method for large-scale detection of antibody-antigen interactions.

Given the large number of input analytes present in PhIP-seq experiments, there is the potential for nonspecific interactions that could result in false positives. This includes non–immunoglobulin-dependent interactions, such as aggregation and protein binding to bead matrices. This problem is further compounded by naturally occurring autoreactivities arising from the unique repertoire of antibodies present in every individual, most of which are unlikely to be associated with disease pathology (48). Recent work has demonstrated that PhIP-seq applications in humans require appropriately large numbers of healthy control samples to eliminate false positives and autoreactivities not associated with disease (11). Model organisms afford unique opportunities to evaluate PhIP-seq performance. For example, using mice that do not produce immunoglobulins, fewer than 10 false-positive autoreactivities were detected compared with mock-IP controls, despite 3 rounds of iterative phage enrichment. This indicates that interactions detected by PhIP-seq primarily arise from immunoglobulin-antigen interactions and not spurious protein-phage binding.

The murine PhIP-seq library was designed with long 62–amino acid overlapping segments corresponding to all the known coding mouse sequences. However, it is well understood that linear epitope binding by antibodies typically involves recognition of relatively short sequences, ranging from 4 to 12 amino acids (49). It is also appreciated that antibodies may vary widely with respect to specificity, including binding of biochemically similar motifs, but nonidentical sequences. This represents both a challenge and an opportunity. For any given collection of PhIP-seq–enriched peptides, the challenge is deducing which motifs within the long phage-displayed peptides are interacting with immunoglobulins, and which peptides that derive from separate coding sequences are in fact being enriched by a shared motif. We have previously shown that motif-finding algorithms, such as MEME (50), may be used to address this challenge by approximating the shared motifs among enriched peptides (13). However, the natural variation of sequences within a proteome is also an opportunity given that many subtle variations of biochemically shared motifs are likely to be inherently embedded in the library. This facet of PhIP-seq was demonstrated using OB1 mice, which possess a predominant high-affinity IgG specificity for chicken OVA (51). OVA is not a murine protein, and thus the precise OVA protein sequence is not represented in the murine library. Despite this fact, serum from these mice enriched over 193 peptides derived from over 133 proteins, in many cases at levels greater than 1000-fold over the background model. Motif analysis of enriched peptides revealed the canonical FGD motif for the known OVA epitope, and further highlighted the importance of a preceding Gly residue that was not previously appreciated. In this manner, epitope-level biochemical motif definitions may arise from PhIP-seq analysis, even in libraries lacking exact matches, in addition to capturing the range of antibody cross-reactivity. This also highlights the complex nature of autoimmune interactions, wherein an antibody thought to be highly specific has the potential to interact with a multitude of similar sequences. While many of these off-target sequences may not be physiologically relevant, we note that off-target interactions have, at the minimum, the potential to complicate interpretations of immune dysregulation.

PhIP-seq has the inherent capacity to yield large numbers of putative autoreactive antigens; however, orthogonal validation by separate assays can be a time-consuming, labor-intensive process. Here, we introduce the SLBA, which is a variant of the RLBA (45) and LIPS (44). With SLBA, oligomers from the PhIP-seq library or full-length proteins can be rapidly fused to a split luciferase tag, which is a compact 11–amino acid sequence, without requiring cloning. The time from synthetic oligomer receipt to IP result can be as fast as 2 days and requires no radioactive reagents. Similar to RLBA and LIPS, this approach may be extended to investigate any protein-protein interaction by co-IP.

To further characterize the murine PhIP-seq library, 3 autoimmune-prone mouse lines were used, specifically *Lyn^–/–^*, NOD, and *Aire^–/–^*. In all 3 models, unique patterns of autoreactivity were identified that corresponded with underlying defects in immune tolerance and reflect what is seen in humans. For example, anti-nuclear and apoptotic reactivities identified in *Lyn^–/–^* mice are consistent with autoantigen array data, previous reports (21–23), and descriptions in lupus patients (24–28). The majority of *Lyn^–/–^*-specific autoreactivities identified by autoantigen protein array were also identified by PhIP-seq, although PhIP-seq identified hundreds of additional reactivities, pointing to a strength of this unbiased, proteome-wide approach. For the 12 proteins that were not detected by PhIP-seq, it may be that the epitopes in these cases are conformational, as opposed to linear. Similarly, we are likely to detect epitopes by peptide display that may be buried or inaccessible on full-length proteins that would only be exposed during processing or degradation, for example.

Some autoimmune diseases exhibit sex-specific traits. We observed autoreactivity to seminal vesicle antigens in male, but not female, NOD mice, consistent with previous reports of prostate-specific lymphocytic infiltration. While we did not have sufficient numbers of female mice to determine this in our study of *Lyn^–/–^* *IgD^+/–^* mice, identifying sex-specific autoreactivities will be critical for this model of lupus disease, which is more prevalent in women.

In the NOD background, murine PhIP-seq analysis revealed enhanced autoreactivity relative to the wild-type B6 background, spanning proteins that derive from multiple tissues, including the pancreas, lung, salivary, and male reproductive tissues. Of note, we did not detect strong enrichment of insulin-reactive autoantibodies in our approach, which is a known autoantibody in the NOD mouse strain (52). This could be related to the improper display of tertiary or secondary structures that are critical for this reactivity and is a known limitation of our approach.

*Aire^–/–^* mice afford an opportunity to investigate an extreme phenotype of autoimmune dysregulation due to a loss of central tolerance. Aire controls medullary thymic epithelial cell (mTEC) expression of self-antigens necessary for negative selection of autoreactive T cells (1). Nearly half of known Aire-dependent proteins expressed in mTECs were identified as autoreactivities in *Aire^–/–^* sera by PhIP-seq. This suggests that failure to negatively select T cells to these proteins results in cognate autoreactivities. These results exemplify the direct relationship between Aire-regulated mTEC antigen expression and resultant antibody autoreactivity. Furthermore, the autoreactivities identified in *Aire^–/–^* mice were highly orthologous to those in APS1 patients, which lack functional *Aire*; the majority of identified autoreactivities in humans were also present in NOD.*Aire^–/–^* mice. These results point to the importance of unbiased proteome-wide autoreactivity profiling in building our understanding of the fundamental relationship of antigen presentation in the thymus and the development of central tolerance in mouse and humans. Looking forward, it will be interesting to see whether other models with defects in central tolerance have an overlap in the array of autoantigens defined here.

The most significantly enriched autoreactive target in *Aire^–/–^* mice was Plin1, whose mTEC expression is also dependent on *Aire* (9, 42). Anti-Plin1 antibodies were recently identified in patients with acquired generalized lipodystrophy, APS1, and cancer immunotherapy treatment (9). We previously demonstrated that archived *Aire^–/–^* mice on the B6 background exhibited anti-Plin1 autoantibodies, and in this study we identified that Plin1 is the most prevalent autoreactivity in NOD.*Aire^–/–^*mice. Plin1 is an intracellular protein essential for lipid storage and lipolysis in adipocytes (53); therefore, it is unlikely that the antibody acts on Plin1 within the cell. It is more plausible that the antibodies against Plin1 are not necessarily directly pathogenic, and the disease is T cell mediated, as has been demonstrated in a number of other *Aire^–/–^*-related autoantigens (54, 55). This scenario would be analogous to paraneoplastic autoimmune disease, like anti-Hu encephalitis, wherein the effectors of inflammation are cytotoxic T cells, reactive to peptides derived from the same proteins to which the B cell response is directed (e.g., anti-HuD) (56). Consistent with this notion, T cell infiltrates were observed in fat pads of *Aire^–/–^* mice. Importantly, these results suggest that the NOD.*Aire^–/–^* mouse model includes the spontaneous development of acquired autoimmune lipodystrophy. To date, no such animal model has been described for this disease to our knowledge and could open new avenues to further understand this important clinical autoimmune disease.

PhIP-seq is a modular and low-cost approach to identify antibody reactivities that may be broadly applied to further define autoimmunity in mouse models beyond the present study and is a powerful complement to traditional antigen screening techniques, such as IP-mass spectrometry. The mouse, as a model organism, affords unique opportunities to identify autoreactivities in a myriad of well-defined immunological perturbations such as immunization, infection, grafts, pregnancies, or tumors.

Our newly defined and validated murine PhIP-seq approach can now be a powerful adjunctive approach to develop a more comprehensive and holistic view of autoreactive landscapes in these important models.

## Methods

### Mouse strains.

Mice were maintained in a specific pathogen–free facility at 25°C, ambient humidity, and a 12-hour light/dark cycle.

Wild-type B6, NOD, and *Rag2^–/–^* mice (B6.Cg-*Rag2^tm1.1Cgn^*/J) (57) mice were purchased from the Jackson Laboratory. Unless otherwise indicated, mutant strains were also on the C57BL/6J background. OB1 transnuclear mice (51) were a gift from Hidde Ploegh at Boston Children’s Hospital (Boston, Massachusetts, USA). *Lyn^–/–^* *IgD*^+/–^ mice have been previously described (17) and serum containing known high titers of autoantibodies were used for experiments. NOD mice were obtained from the Jackson Laboratory and NOD.*Aire^–/–^* mice have been previously described (58). NOD control and *Aire^–/–^* mice were greater than 12 weeks of age.

Serum was obtained via orbital bleed and allowed to clot for 45 minutes at room temperature, and then centrifuged at 1500*g* for 10 minutes to collect the serum supernatant, which was promptly aliquoted and frozen for temporary storage prior to experimentation.

### Library construction, cloning, and sequence validation.

*Mus musculus* reference proteome sequences, including all isoforms (GRCm38.p5) were downloaded from the NCBI and fragmented into 62–amino acid peptide tiles with 19–amino acid overlap. Peptides were clustered to 95% similarity and low-complexity sequences were removed with the Lempel-Ziv-Welch algorithm. The resulting library of 482,672 peptides was synonymously scrambled to prevent self-annealing at overlapping regions, codon-optimized for *Escherichia coli*, and synthesized as an oligomer pool (Agilent Inc). Each peptide contained an alanine/glycine N-terminal linker (AAVVGGV) and a double-stop C-terminal linker (**AYAMA). The resultant oligomer library was cloned into a T7 phage display system (Millipore) by using primers to attach restriction enzyme sites, ligated into the T7 genome, and packaged into phage, as previously described (13).

Linker-specific dephasing primers were used to amplify the genomic insert corresponding to the displayed peptide from the synthesized oligomer pool and library after cloning. Amplicon libraries were indexed for next-generation sequencing using the NovaSeq 6000 platform (Illumina), as previously described (13). A step-by-step library construction protocol is available at https://www.protocols.io/view/mouseome-cloning-and-phip-seq-protocol-kxygx9144g8j/v1

### PhIP-seq with murine serum.

PhIP-seq was performed as previously described (8, 13) by incubating 0.5 mL of mouse sera and 0.5 mL 2× antibody storage buffer (40% glycerol, 40 mM HEPES pH 7.3, 0.04% [w/v] NaN_3_, 2× PBS) with 1 mL of murine PhIP-seq library at approximately 1 × 10^11^ plaque forming units (PFU)/mL with overhead rotation overnight at 4°C. Bound immune complexes were captured with an equal mix of protein A and protein G Dynabead magnetic beads (Thermo Fisher Scientific), which recognize all mouse IgG subclasses, washed to remove nonspecific binding, and inoculated into *E*. *coli* cultures for lytic phage amplification. Three successive rounds of amplification were performed for each experiment and the last 2 rounds were sequenced for each experiment. Each serum was tested in at least technical duplicate. Mock IPs were performed after incubating the murine PhIP-seq library with 1 μL of antibody storage buffer in lieu of serum, but otherwise followed the same IP and amplification protocol. Positive control PhIP-seq designed to capture mouse and human GFAP began by incubating 0.1 μg/mL polyclonal anti-GFAP antibody (Dako, catalog Z0334) with the murine PhIP-seq library.

### Anti-nuclear antibody staining.

Anti-nuclear immunofluorescent staining was performed as previously described (23). Briefly, Kallestad HEp-2 slides (Bio-Rad) were stained overnight with 1 μL of serum from either *Lyn^–/–^* *IgD*^+/–^ or wild-type mice. After washing, antibody binding was detected using an anti-mouse secondary antibody conjugated to Alexa Fluor 488 (Thermo Fisher Scientific) and DAPI counterstain (Thermo Fisher Scientific). Cells were imaged with the Crest LFOV Spinning Disk/C2 confocal microscope at ×1000 magnification under identical camera exposure and laser settings for knockout and control mice. Micrograph exposure was normalized to secondary-only negative control in FIJI and applied to all images at once.

### Identification of Lyn^–/–^ reactivities using an autoantigen array.

Serum was shipped to the University of Texas Southwestern microarray core facility for analysis, as previously described (59). Autoantigen array results were filtered for protein antigens, excluding nonprotein antigens, complex antigens, or posttranslationally modified antigens. Lyn-specific antigens were identified by utilizing the average signal from IgG-normalized data. Means and standard deviation from wild-type mice were used to calculate *z* scores for *Lyn^–/–^* mice and an antigen was considered to be a hit if it met a *z*-score threshold of 3 and Lyn-specific if more than 2 *Lyn^–/–^* mice had the peptide as a hit while none of the wild-type mice did. Lyn-specific autoreactivities identified by autoantigen array were compared to autoreactivities identified by PhIP-seq in *Lyn^–/–^* *IgD*^+/–^ mice by comparing lists of hit peptides.

### SLBA.

A detailed SLBA protocol is available on protocols.io at https://www.protocols.io/view/split-luciferase-binding-assay-slba-protocol-4r3l27b9pg1y/v1 Briefly, peptides tiling across Plin1 were inserted into a split luciferase construct containing a terminal HiBiT tag and synthesized (Twist Biosciences) as DNA oligomers. Constructs were amplified by PCR using 5′-AAGCAGAGCTCGTTTAGTGAACCGTCAGA-3′ and 5′-GGCCGGCCGTTTAAACGCTGATCTT-3′ primer pair. Unpurified PCR product was used as input to a rabbit reticulocyte transcription translation system (Promega) and Nano-Glo HiBit Lytic Detection System (Promega, N3040) was used to measure relative luciferase units (RLU) of translated peptides in a luminometer. Peptides were normalized to 2 × 10^7^ RLU input, incubated overnight with mouse sera, and immunoprecipitated with protein A and protein G sepharose beads (MilliporeSigma). After thoroughly washing beads with SLBA buffer (0.15 M NaCl, 0.02 M Tris-HCl pH 7.4, 1% w/v sodium azide, 1% w/v bovine serum albumin, and 0.15% v/v Tween 20), luminescence remaining on beads was measured using Nano-Glo HiBit Lytic Detection System (Promega, N3040) in a luminometer. Anti-HiBiT antibody (Promega) was used as a positive control for each peptide. Antibody index was calculated by dividing the RLU for each experimental condition by the RLU obtained by anti-HiBiT antibody. Fold-change in *Aire^–/–^* mice was calculated by dividing the antibody index by the mean antibody index in wild-type control mice.

### Adipose tissue microscopy and quantification.

Perigonadal fat pads were dissected, fixed in 10% neutral buffered formalin for 24 hours, and sent for processing to Histowiz for H&E staining as well as immunohistochemistry. Images were quantified in QuPath software (60) using the “cell detection” tool within a rectangular region of interest. The number of detected cells was normalized per 1 × 10^7^ μm^2^ area.

### Bioinformatics analysis of PhIP-seq data.

Next-generation sequencing reads from synthesized or cloned oligomer libraries were aligned to the designed library with bowtie2 (61), as previously described (8). To analyze peptide enrichment after PhIP-seq, reads were aligned at the protein level using RAPsearch (62), as previously described (8). Aligned reads were normalized to 100,000 reads per k-mer (RPK) to account for varying read-depth and further normalized to the mean of *Rag2^–/–^*, μMT, and mock IP controls within each experimental batch. A postnormalization batch effect was not observed. Autocorrelation between technical replicated samples was evaluated by Pearson’s correlation. RPK was averaged across technical replicates and strain-specific candidate antigens were identified using an implementation of the PhagePy python package (https://github.com/h-s-miller/phagepy). Briefly, a *z* score of greater than or equal to 3 and fold change greater than 2 over background were required in test strain versus control. For experiments evaluating library performance relative to mock IP, per-animal *z* scores were calculated for each peptide and filtered as above.

To assess the off-target binding of OB1 sera to peptides in the murine PhIP-seq library, every peptide in the library was scanned for sequence similarity to the OVA epitope DKLPGFGDSI using FIMO (63), with a *P*-value threshold of less than 0.0001. Peptides in the murine proteome with significant sequence similarity to the OVA epitope were filtered by presence of the essential FGD sequence. Epitope scoring was developed to evaluate peptide enrichment of the FGD motif as follows. Each peptide was scanned in a 4-mer sliding window and scored in each 4-mer window. The score was defined as 500 if the 4-mer contained the essential epitope FGD or else given by the Smith-Waterman alignment score between FGD and the 4-mer. The Smith-Waterman algorithm (implemented with scikit-bio; https://scikit.bio/) used a substitution matrix in which amino acid matches were scored 25, amino acid substitutions within Dayhoff category were scored 10, and amino acid substitutions outside of Dayhoff category were scored –25 and used a gap open penalty of 1 and gap extension penalty of 4. The total alignment score was taken to be the summation of scores across all 4-mer windows.

Human orthologs of NOD-specific peptides were surveyed for tissue expression in the salivary gland, seminal vesicle, prostate, pancreas, and lung using tissue expression data from the human protein atlas (https://www.proteinatlas.org/about/download Accessed August 1, 2023.). Peptides were labeled as “expressed” in the given tissue type if their protein ortholog had “high” level of expression as determined by immunohistochemistry of tissue microarrays.

### Statistics.

The Kruskal-Wallis test with Dunn’s multiple-comparison was used to evaluate significance between fold changes for selected proteins or pathways across mouse strains, unless otherwise indicated in the figure legend. A *P* value of less than 0.05 was considered significant. Data are presented as mean ± SEM.

### Study approval.

Animal experiments were conducted under IACUC-approved protocols at UCSF.

### Data availability.

All supporting data for each figure panel are available in the Supporting Data Values file. Design files for the murine PhIP-seq library, as well as raw and aligned PhIP-Seq data and all underlying data associated with this manuscript are available for download at Dryad (https://doi.org/10.7272/Q6BG2M7B). Analytical code is freely available on GitHub: https://github.com/h-s-miller/phagepy

## Author contributions

ER, IP, GR, and JLD designed the research. ER, IP, HSM, JFB, CMB, DY, CRZ, RB, SEV, SS, CLA, and GR performed the research. ER, HSM, AR, AFK, CMB, GR, and JLD contributed to new reagents/analytic tools. ER, IP, HSM, CAL, MRW, JZ, MSA, and JLD analyzed data. ER and JLD wrote the manuscript. All authors reviewed the manuscript and provided feedback.

## Supplementary Material

Supplemental data

Supplemental tables 1-5

Supporting data values

## Version 1 11/07/2023

In-Press Preview

## Version 2 12/08/2023

Electronic publication
