# Supplementary material for: Validation of a murine proteome-wide phage display library for identification of autoantibody specificities
Source: JCI Insight. 2023 Dec 8;8(23):e174976. doi: 10.1172/jci.insight.174976 (PMC10795829; doi:10.1172/jci.insight.174976)
Supplement: Supplemental data [file jciinsight-8-174976-s103.pdf]

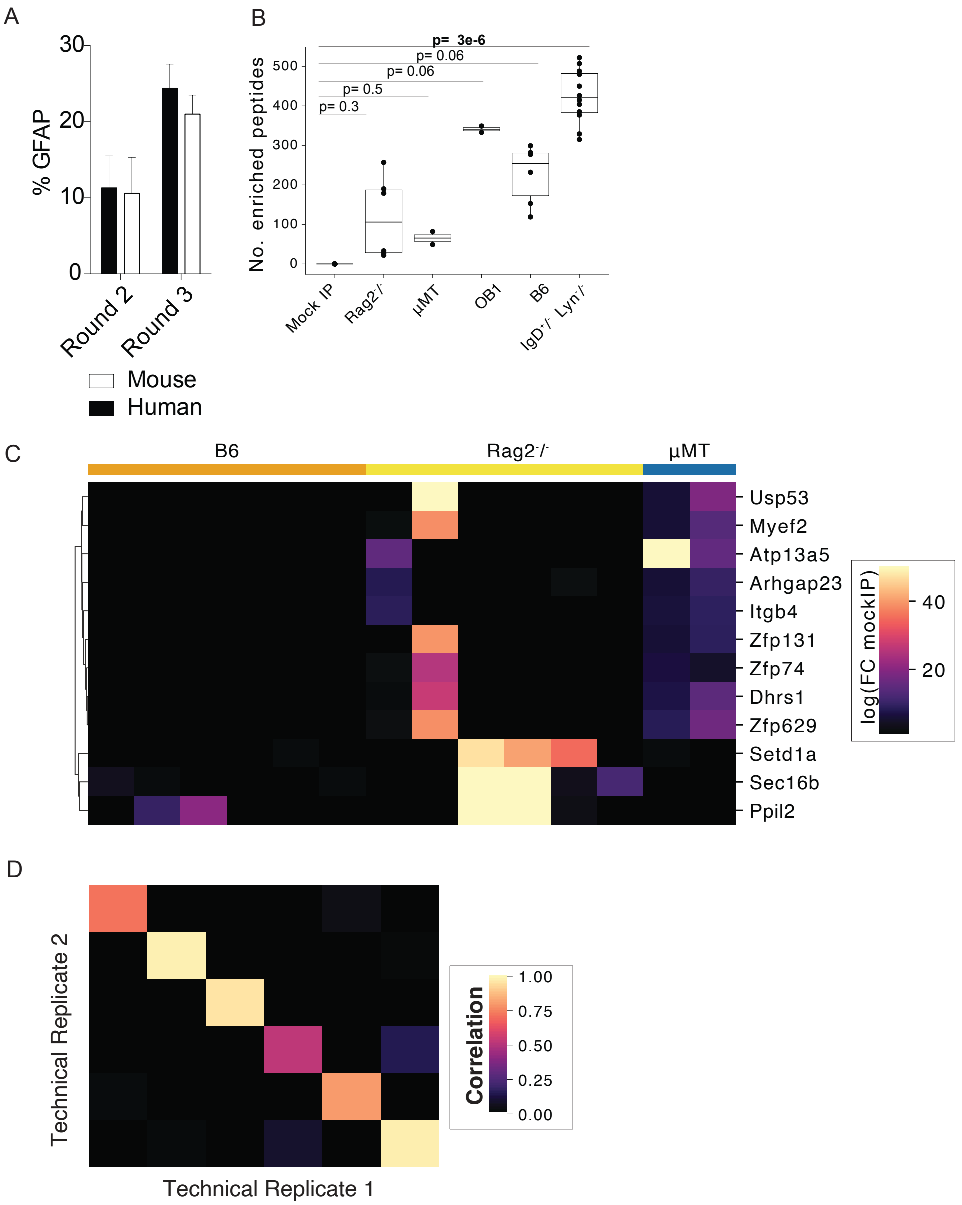

Supplementary Figure 1. Background modeling with *Rag2*$^{-/-}$ and µMT mice. A. Percent of reads mapping to human and mouse GFAP positive control peptides after two and three rounds of panning. **B.** Number of enriched peptides identified with a z-score greater than 3 and fold-change greater than 2 compared to mockIP in each experimental sample (mock IP) or mouse strain (*Rag2*$^{-/-}$, µMT, OB1, B6, and *IgD*$^{+/-}$ *Lyn*$^{-/-}$). **C.** Heatmap of top peptide log 10 fold change over mock IP in *Rag2*$^{-/-}$ or µMT mice compared to B6 mice. Exact p-value is reported, each dot corresponds to a mouse or mockIP replicate; Kruskal Wallis test with Dunn post-hoc. **D.** Heatmap of Pearson correlation of two technically replicated peptide enrichments in *Lyn*$^{-/-}$ *IgD*$^{+/-}$ mice by PhIP-seq.

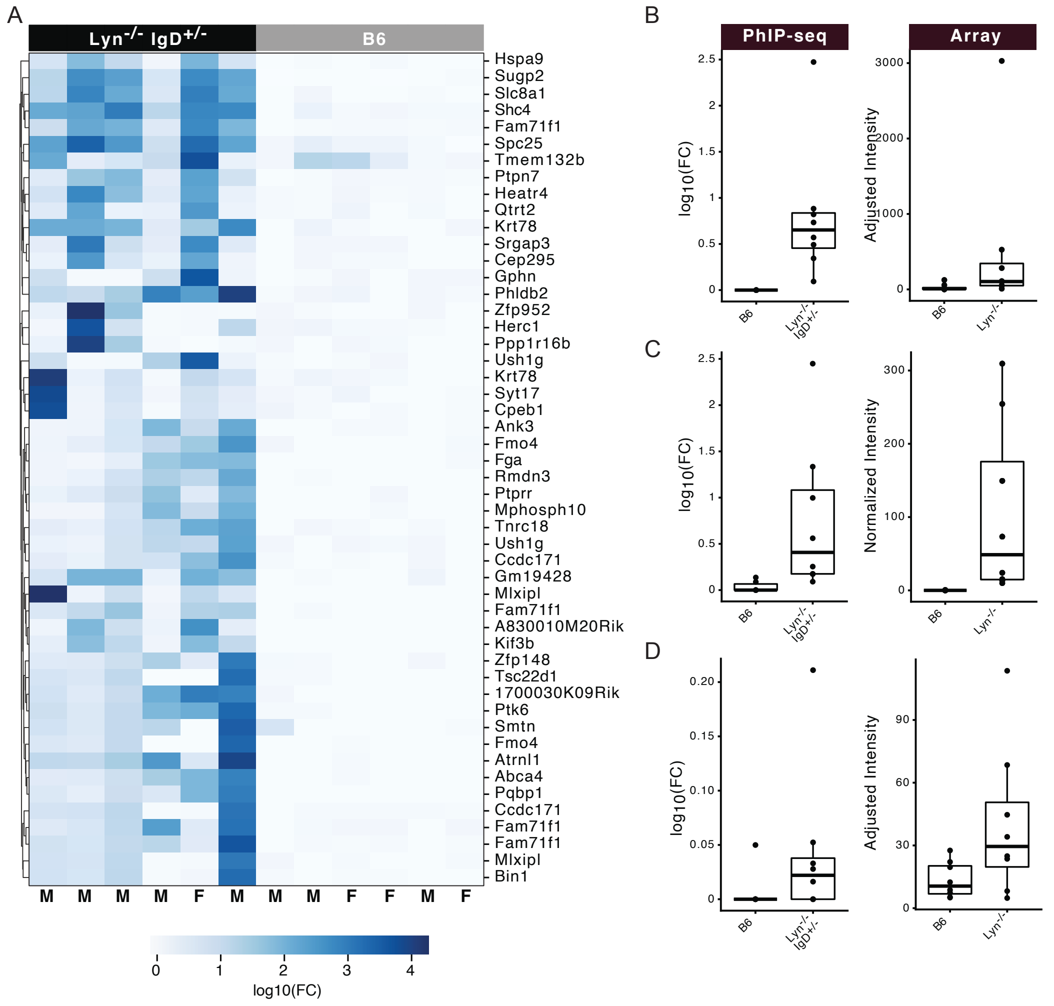

Supplementary Figure 2. **Autoreactive peptides in *Lyn$^{-/-}$ IgD$^{+/-}$* mice.** Heatmap of $\log_{10}$ fold change over MBM in **A.** top 50 peptides ranked by fold change in *Lyn$^{-/-}$ IgD$^{+/-}$* versus B6 mice. Peptide enrichments were identified by PhIP-seq in A and annotated by their corresponding protein. Male (M) or female (F) mouse sex indicated on x-axis. Log_{10} fold change over MBM or normalized intensity of **B.** Snrp/SmD **C.** Collgen VI, **D.** or Laminin in *Lyn$^{-/-}$IgD$^{+/-}$* (left) or *Lyn$^{-/-}$* (right) or wildtype control mice as detected by PhIP-seq (left) or autoantigen array (right).

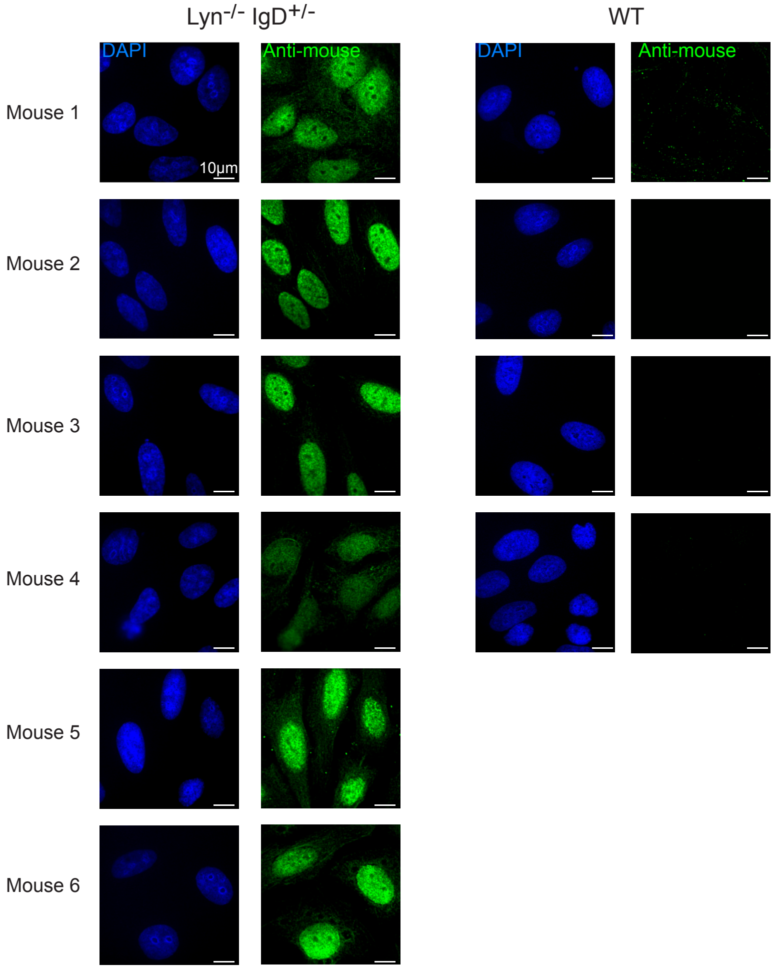

Supplementary Figure 3. Anti-nuclear antibodies in *Lyn*$^{-/-}$ *IgD*$^{+/-}$ mice. Representative images of immunofluorescence detection of *Lyn*-$^{/-}$*IgD*$^{+/-}$ (left) or wildtype (right) mouse sera binding to nuclei of HEp-2 cell line. Side-by-side single channel emission for DAPI and anti-mouse Alexa488 is shown for each mouse. Scale bar corresponds to 10µm.

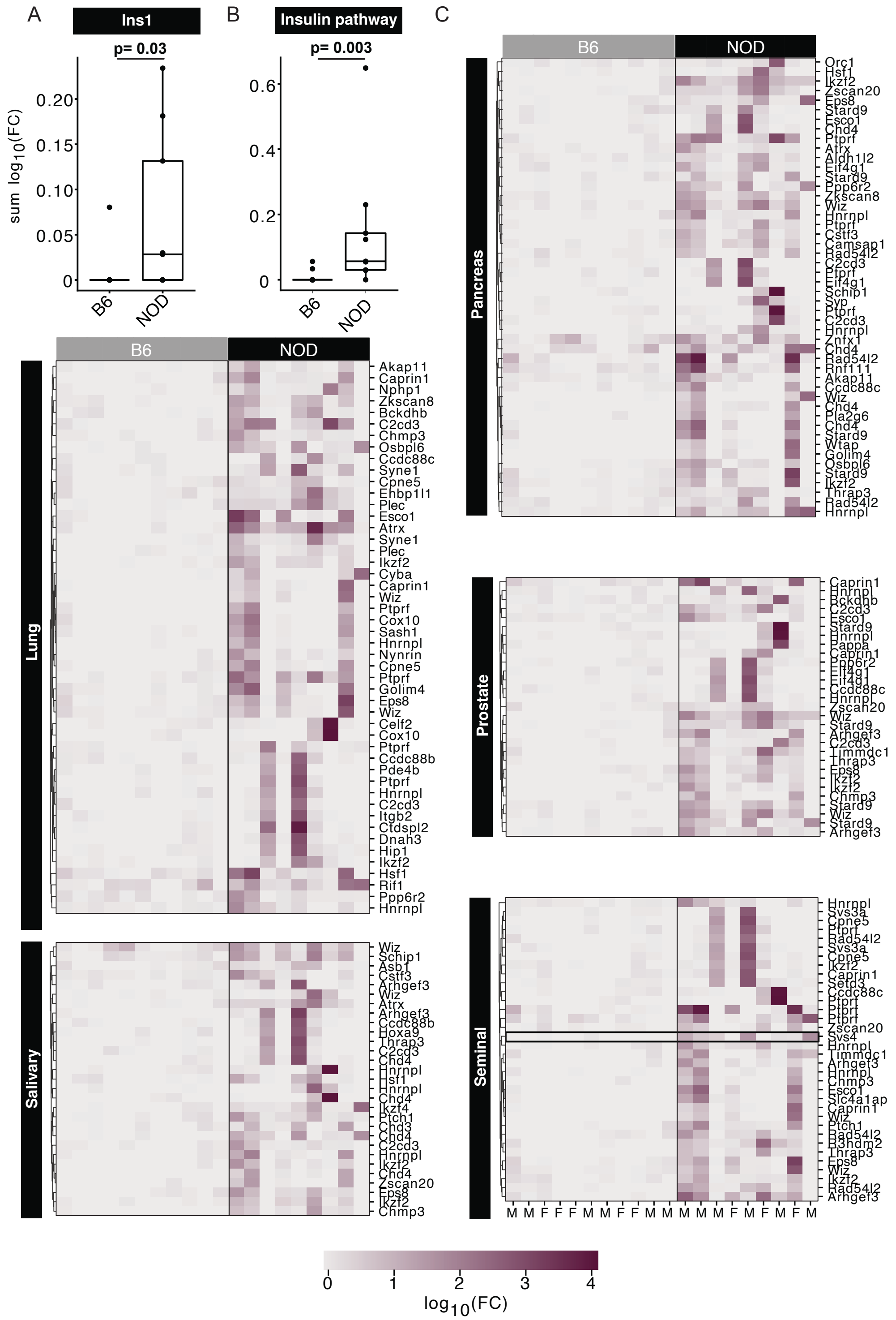

Supplementary Figure 4. Autoreactive peptides in NOD mice. A. Sum $\log_{10}$ fold change over MBM and **B-C**. heatmap of $\log_{10}$ fold change of insulin pathway (A) or predicted pancreatic proteins (B) or 50 peptides ranked by fold change (C) in B6 or NOD mice. Male (M) or female (F) mouse sex indicated on x-axis in C. Peptide enrichments were identified by PhIP-seq in and annotated by their corresponding protein.

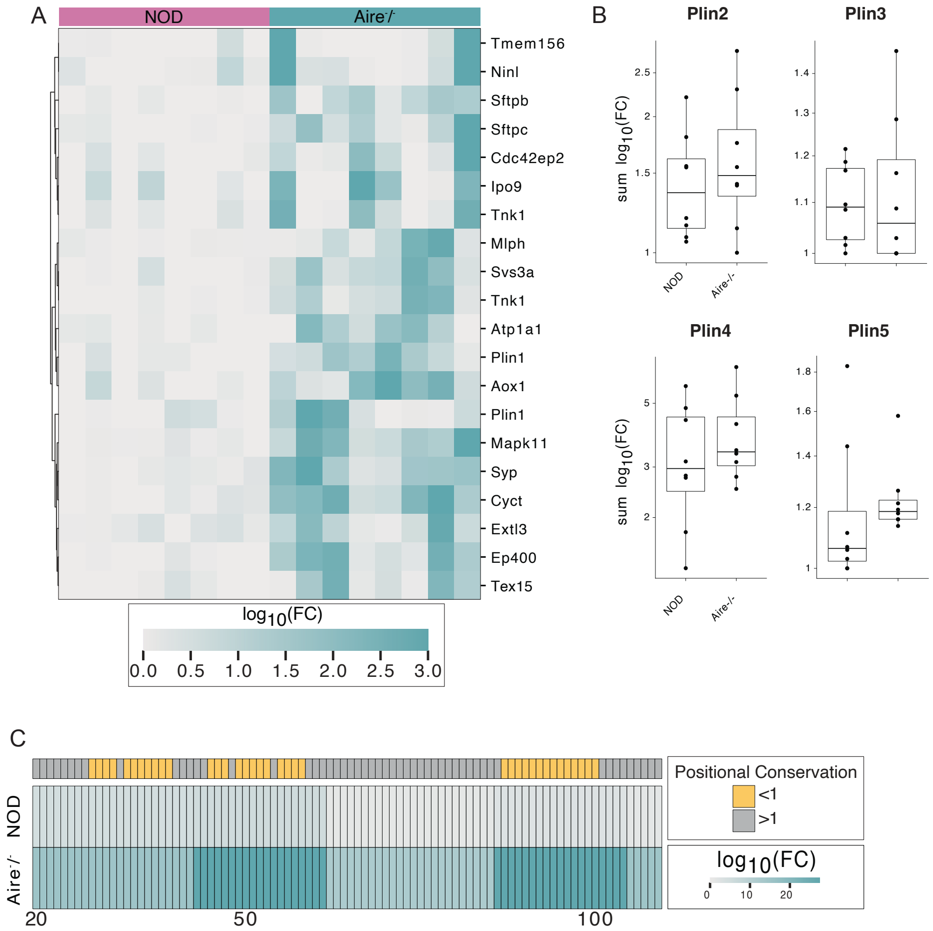

Supplementary Figure 5. Autoreactive peptides in *Aire*$^{-/-}$ mice. A. Heatmap of $\log_{10}$ fold change over background in top 20 peptides ranked by fold change in *Aire*$^{-/-}$ versus NOD mice. **B.** Sum $\log_{10}$ fold change over MBM of peptides tiling across Plin2, Plin3, Plin4, and Plin5. **C.** Heatmap of Plin1 PAT domain positional sum $\log_{10}$ fold change over MBM in *Aire*$^{-/-}$ or NOD mice annotated with positional conservation with other perilipin family proteins. Peptide enrichments were identified by PhIP-seq and annotated by their corresponding protein.

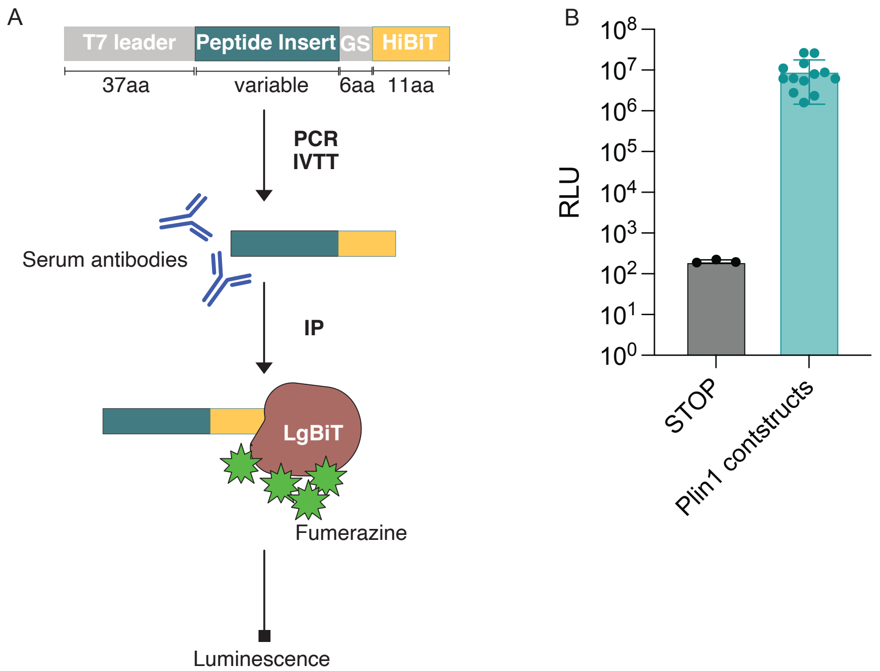

Supplementary Figure 6. Method for split luciferase binding assay. A. Schematic of split luciferase binding assay (SLBA) protocol. HiBiT-tagged constructs are synthesized as DNA oligomers, amplified by PCR, and *in vitro* translated (IVTT). After immunoprecipitation (IP) with serum antibodies, peptide enrichment is quantified by adding LgBiT that complexes with HiBiT tag and generates luminescence given a fumerazine substrate. **B.** Relative luminescence units (RLU) of in-frame stop codon or Plin1 constructs.

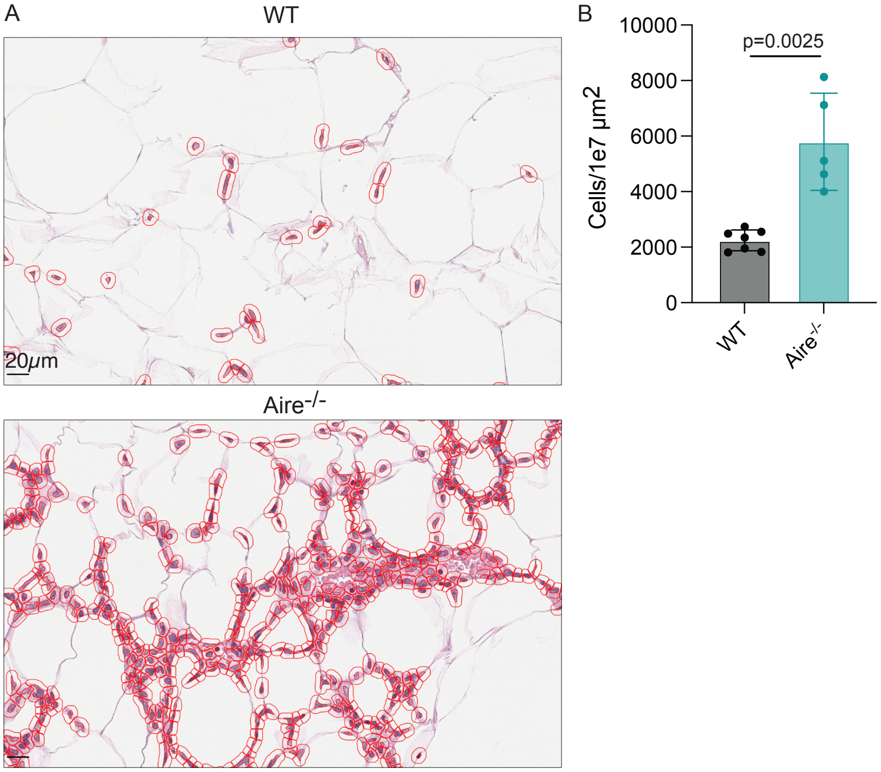

Supplementary Figure 7. Quantification of cell-infiltrates in adipose tissue of *Aire*$^{-/-}$ mice. A. Representative images of inguinal fat pads stained with H&E and infiltrating cell boundaries (red) identified by QuPath software in wildtype NOD or *Aire*$^{-/-}$ NOD mice. Scale bar corresponds to 20μm. **B**. Infiltrating cells per 1e7μm^2 area in wildtype NOD or *Aire*$^{-/-}$ NOD mice. KS test for significance.
